# Genome Sequences of Human Coronavirus OC43 and NL63, Associated with Respiratory Infections in Kilifi, Kenya

**DOI:** 10.1128/MRA.00730-19

**Published:** 2019-11-14

**Authors:** Everlyn Kamau, Martha M. Luka, Zaydah R. de Laurent, Irene Adema, Charles N. Agoti, D. James Nokes

**Affiliations:** a KEMRI-Wellcome Trust Research Programme, Kilifi, Kenya; b Department of Public Health, Pwani University, Kilifi, Kenya; c School of Life Sciences and Zeeman Institute SBIDER, University of Warwick, Coventry, United Kingdom; KU Leuven

## Abstract

Coding-complete genomes of two human coronavirus OC43 strains and one NL63 strain were obtained by metagenomic sequencing of clinical samples collected in 2017 and 2018 in Kilifi, Kenya. Maximum likelihood phylogenies showed that the OC43 strains were genetically dissimilar and that the NL63 strain was closely related to NL63 genotype B viruses.

## ANNOUNCEMENT

Human coronaviruses (HCoVs) are enveloped viruses within the family *Coronaviridae* with positive-sense, single-stranded genomes of up to 32 kb, with a 5′ cap structure and 3′ polyadenylation tract (1). The 5′ two-thirds of the genome encodes the large replicases 1a and 1b encoding nonstructural proteins, whereas the 3′ one-third encodes the structural proteins (1). Coronaviruses are divided into four genera, namely, *Alphacoronavirus* (229E and NL63), *Betacoronavirus* (severe acute respiratory syndrome [SARS], Middle East respiratory syndrome [MERS], OC43, and HKU1), *Gammacoronavirus*, and *Deltacoronavirus* (2). The NL63, 229E, HKU1, and OC43 species are endemic in human populations, causing relatively mild and severe respiratory disease (2). Up to eight distinct HCoV-OC43 genotypes (3) and three HCoV-NL63 genotypes (4) have been identified. However, there is a paucity of HCoV genomes that have been collected globally, with a negligible number from Africa.

Here, we report coding-complete genomes of two HCoV-OC43 strains and one NL63 strain. The isolates NL63_KLF_01_2018 and OC43_KLF_01_2018 were obtained from acute respiratory infection cases at a local school (from a 7-year-old boy with cough, nasal discharge, and abdominal pain and from a 13-year-old female student with cough, nasal discharge, and fever, respectively). OC43_KLF_02_2017 was isolated from a 2-month-old boy presenting at the Kilifi County Hospital with fever and chest in-drawing. Written informed consent for study participation was obtained from parents or guardians of the patients, and ethical approval was obtained from the KEMRI Scientific and Ethics Review Unit.

Viral RNA was extracted from nasopharyngeal swabs using TRIzol LS reagent (Invitrogen) followed by TURBO DNase treatment (Invitrogen), according to the manufacturer’s instructions. cDNA was synthesized with SuperScript III reverse transcriptase (Invitrogen) with random hexamers, and double-stranded cDNA was synthesized with Klenow polymerase (5 U; New England BioLabs). Libraries were prepared using the Nextera XT kit (Illumina) according to the manufacturer’s instructions and sequenced using the MiSeq reagent kit v2 (500 cycle) (Illumina) on an Illumina MiSeq sequencer.

Sequencing reads (paired, 2 × 250 bp) were filtered, kmer normalized, and *de novo* assembled as previously described (5) by using the settings specified in Table 1. Reference OC43 and NL63 genomes (GenBank accession numbers AY391777 and NC_005831, respectively) were used to transfer annotations to assembled contigs using Geneious (R9). MAFFT v.7.221 (6) was used for sequence alignment using the parameters “–localpair –maxiterate 1000.” Maximum likelihood phylogenies were estimated in RAxML v.8 (7) using the general time-reversible (GTR) nucleotide substitution model and gamma distribution of among site rate variation.

**TABLE 1:** Characteristics of the HCoV OC43 and NL63 genomes and samples from Kilifi, Kenya^*a*^

Isolate	Genome size (nt)	G+C content (%)	No. of reads^*b*^	*N*_50_ (bp)^*c*^	Avg depth (×)^*d*^	No. of contigs	rRT-PCR^*e*^ *C_T_* value	GenBank accession no.
NL63_KLF_01_2018	27,125	34.5	754,456	19,834	348.596	127	20.88	MN026166
OC43_KLF_01_2018	30,777	36.6	622,954	24,566	502.009	240	20.18	MN026164
OC43_KLF_02_2017	30,318	36.6	1,072,826	27,724	983.988	2,219	21.07	MN026165

a QUASR parameters, -d –q –l 125 –m 30; SPAdes parameters, —careful -k 77, 99, 127; QUAST parameters, minimum contig length, 500, ambiguity, one, and threshold for extensive misassembly size, 1,000, bowtie2 parameters, -q –S –local. nt, nucleotide; rRT-PCR, real-time reverse transcription-PCR; *C_T_*, threshold cycle.

b Short read length of the raw sequence data ranged from 35 to 250 bases.

c *N*_50_ length is calculated by summing the lengths of contig assembly of a particular sample from the longest to the shortest and determining the point at which 50% of the assembly size is reached.

d Calculated by dividing the per-position coverage output (described in the text) by respective genome length.

e The rRT-PCR assay, including primers and probe sequences used for HCoV detection, has been described previously (9).

**FIG 1:**
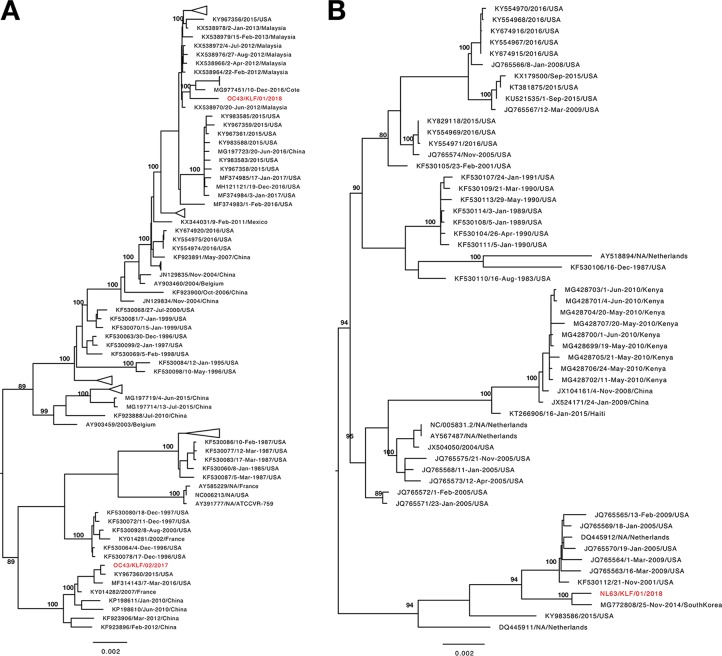
Maximum likelihood phylogenetic tree of 175 OC43 (A) and 56 NL63 (B) genome sequences. Reference and contemporary sequences were retrieved from GenBank. Sequences from Kilifi, Kenya, are colored red. Only nonoverlapping bootstrap values of >70 are shown in the phylogeny. Scale bars indicate expected nucleotide substitutions per site.

Characteristics of the three coronaviruses are listed in Table 1, and their genome organization was typical of NL63 and OC43 (8). Other viral contigs belonging to respiratory syncytial virus (RSV), adenovirus, influenza, and rhinovirus were identified using the BLASTn program, albeit in low frequencies (<0.1% of total contigs per sample). The two OC43 genomes had 98.4% (28,654 sites) pairwise identity but clustered in distinct branches of the genome-based phylogeny (Fig. 1A), while the NL63 sequence grouped with global NL63 genotype B sequences (Fig. 1B). Extensive variability, including insertions, deletions, and nonsynonymous substitutions, was observed in the S gene between Kilifi and other global strains for both NL63 and OC43 viruses. Furthermore, the number of codons and the pattern of putative *N*-glycosylation (N-X-S/T) varied in the spike protein between the two Kilifi OC43 strains. These new complete-coding genomes increase the available data from Africa and will be useful for future molecular epidemiology studies.

### Data availability.

The raw sequence data were deposited in the Sequence Read Archive (SRA) under BioProject accession number PRJNA547576 and BioSample accession numbers SAMN11969662, SAMN11969663, and SAMN11969664. The genome sequences generated here were deposited in GenBank under accession numbers MN026164, MN026165, and MN026166.
